# A Case of Long-term Survival of 36 Months in the Setting of Extensive-disease Small-cell Lung Cancer

**DOI:** 10.7759/cureus.5605

**Published:** 2019-09-09

**Authors:** Gowthami Ramineni, Bikramjit S Bindra, Karan Jatwani, Dilbagh Singh, Ratesh Khillan

**Affiliations:** 1 Internal Medicine, Rajiv Gandhi Institute of Medical Sciences, Ongole, IND; 2 Internal Medicine, Government Medical College and Hospital, Chandigarh, IND; 3 Internal Medicine, Mount Sinai St. Luke's Roosevelt Hospital Center, New York, USA; 4 Internal Medicine, American University of Antigua, Osbourn, ATG; 5 Hematology / Oncology, Kingsbrook Jewish Medical Center, New York, USA

**Keywords:** smoking, small cell lung cancer, checkpoint inhibition

## Abstract

Small-cell lung cancer (SCLC) is an extremely aggressive disease characterized by early regional spread and distant metastases. Patients with extensive-disease (ED) SCLC have a median survival rate of 8-11 months. Despite high response rates to initial therapy, relapses are frequent. Systemic therapy after the first-line failure remains vital in the treatment paradigm of SCLC. The National Comprehensive Cancer Network (NCCN) guidelines dictate that previously administered first-line chemotherapy can be used in relapses that occur after six months from the completion of initial therapy. For relapses within six months of initial therapy, sequential treatment with single agents is recommended. In this report, we discuss the case of a long-term SCLC survivor with an ED. The patient underwent several lines of chemotherapy and prophylactic cranial irradiation (PCI) and survived for 36 months.

## Introduction

Small-cell lung cancer (SCLC) is the most common neuroendocrine (NE) tumor of the lung, which constitutes 20% of all lung cancers [1, 2]. SCLC is strongly associated with smoking, and it is a highly aggressive disease with a propensity for early distant metastasis and paraneoplastic syndromes. The typical survival rate of SCLC is measured in months. The prognosis is dictated by multiple factors including, but not limited to, the extent of the disease, performance status of the patient, weight loss, response to initial treatment, and the severity of the distant metastasis. The five-year survival rate for extensive-disease small-cell lung cancer (ED-SCLC) is 1-2%, and the median range of survival is only 8-13 months [3].

## Case presentation

**Figure 1:**
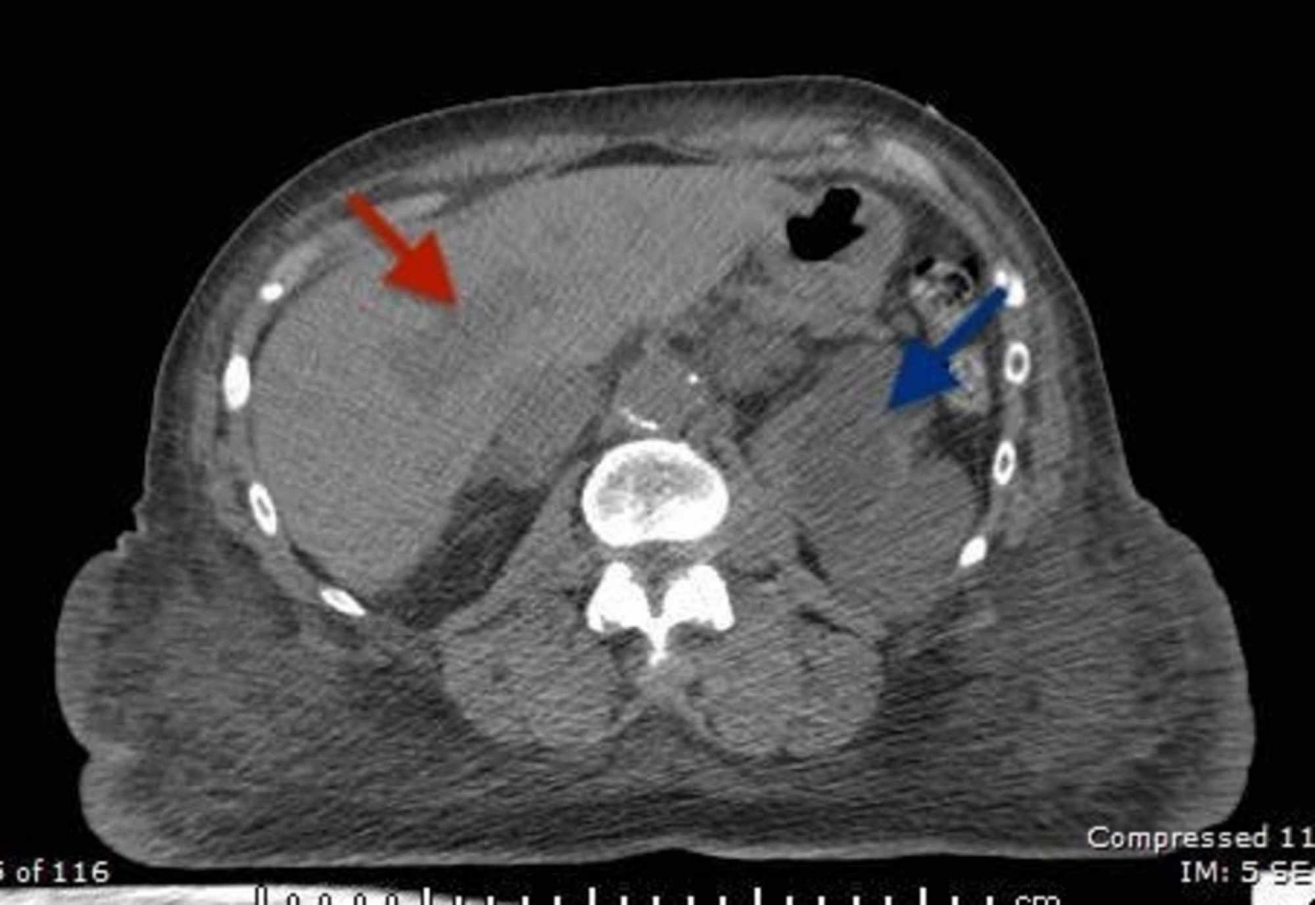
Axial CECT (contrast-enhanced computed tomography) scan of the abdomen showing metastasis to the liver (red arrow) and left adrenal gland (blue arrow).

**Figure 2:**
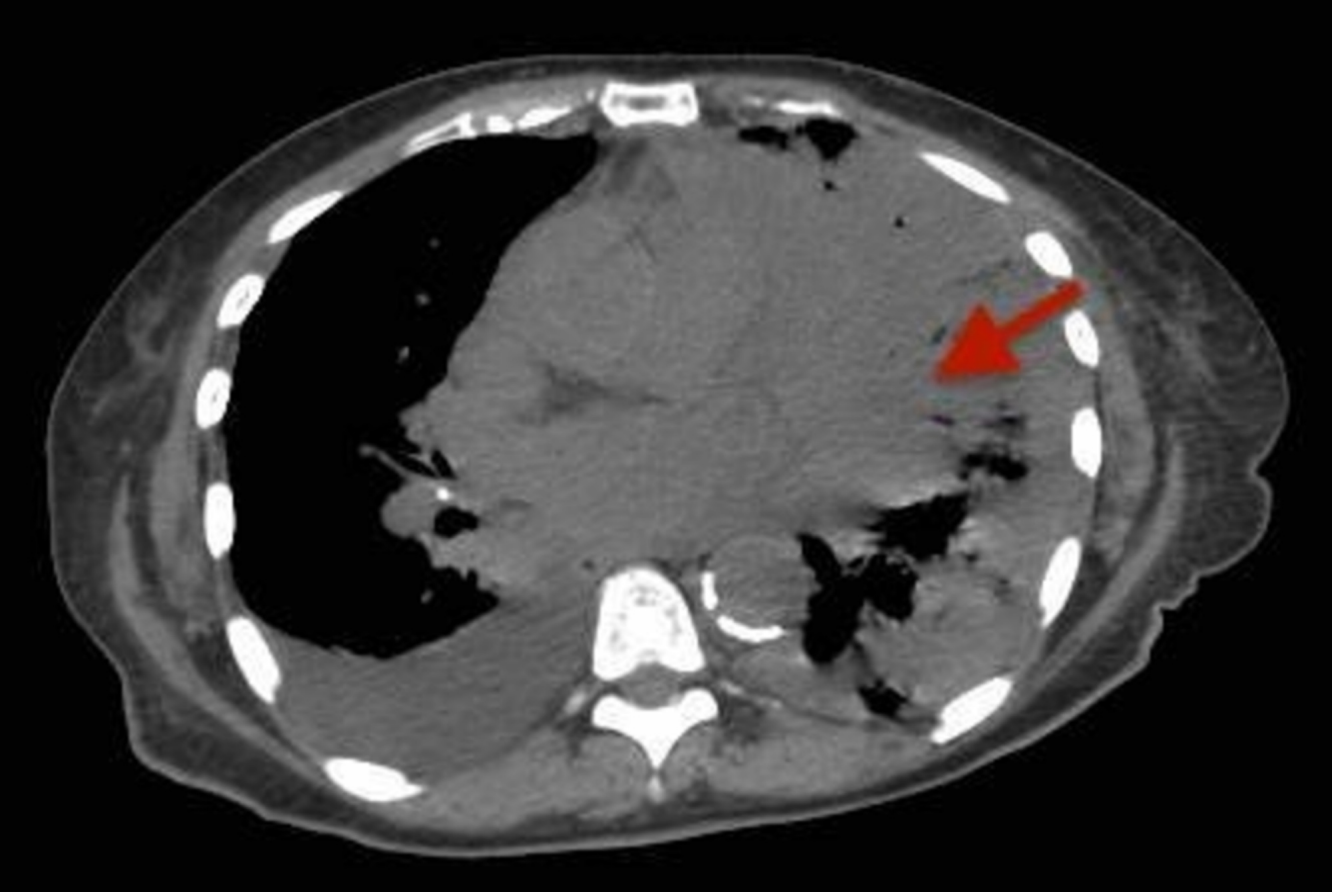
Axial CECT (contrast-enhanced computed tomography) scan of the chest showing a left lung mass extending into the lower lobe (red arrow).

**Figure 3:**
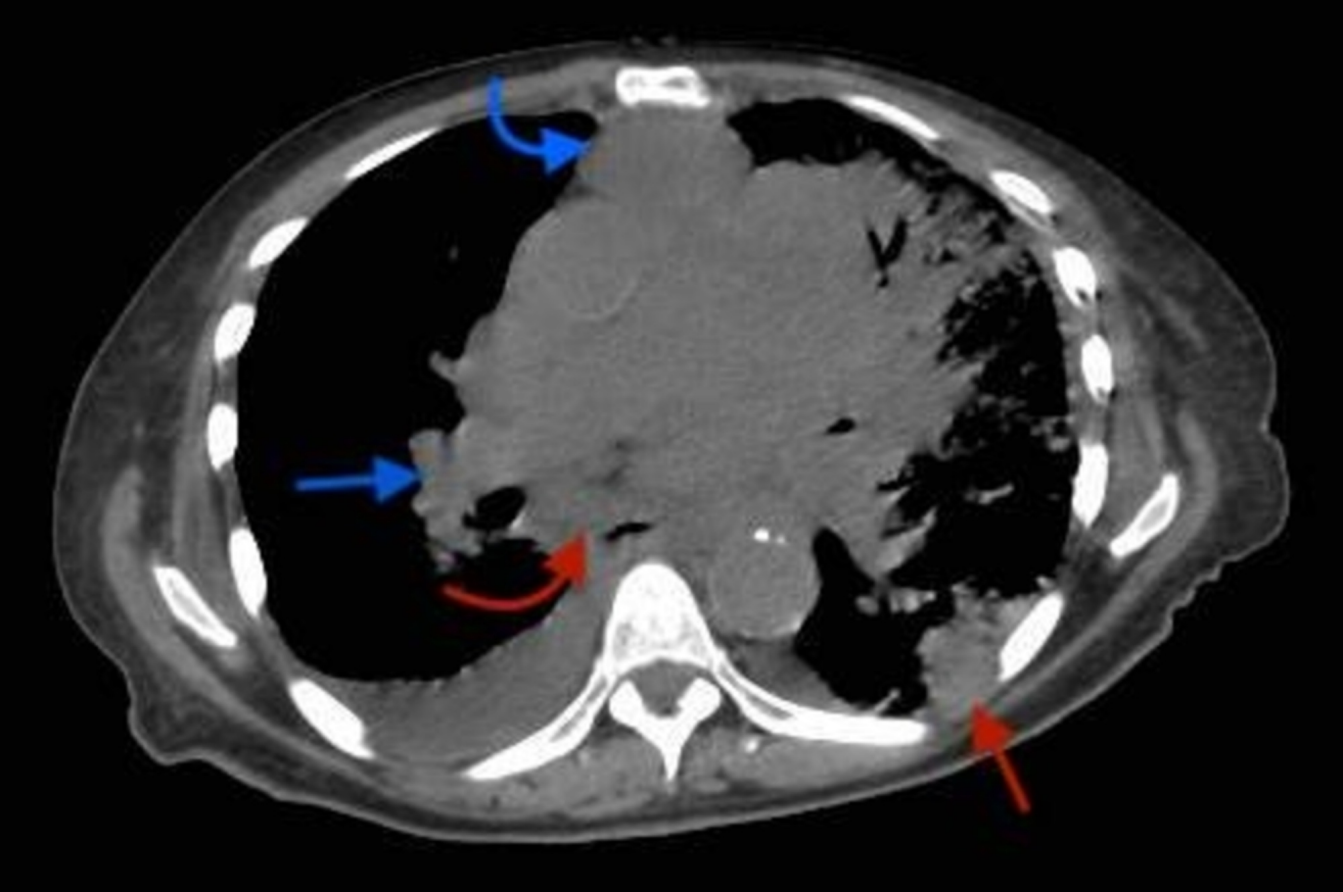
Axial CECT (contrast-enhanced computed tomography) scan at T6 level showing metastatic focus in basal part of right upper lobe with right hilar lymph nodal enlargement (straight blue arrow), enlarged retrosternal/anterior mediastinal lymph node (curved blue arrow), enlarged para-esophageal lymph node (curved red arrow), metastatic pleural involvement (straight red arrow).

**Figure 4:**
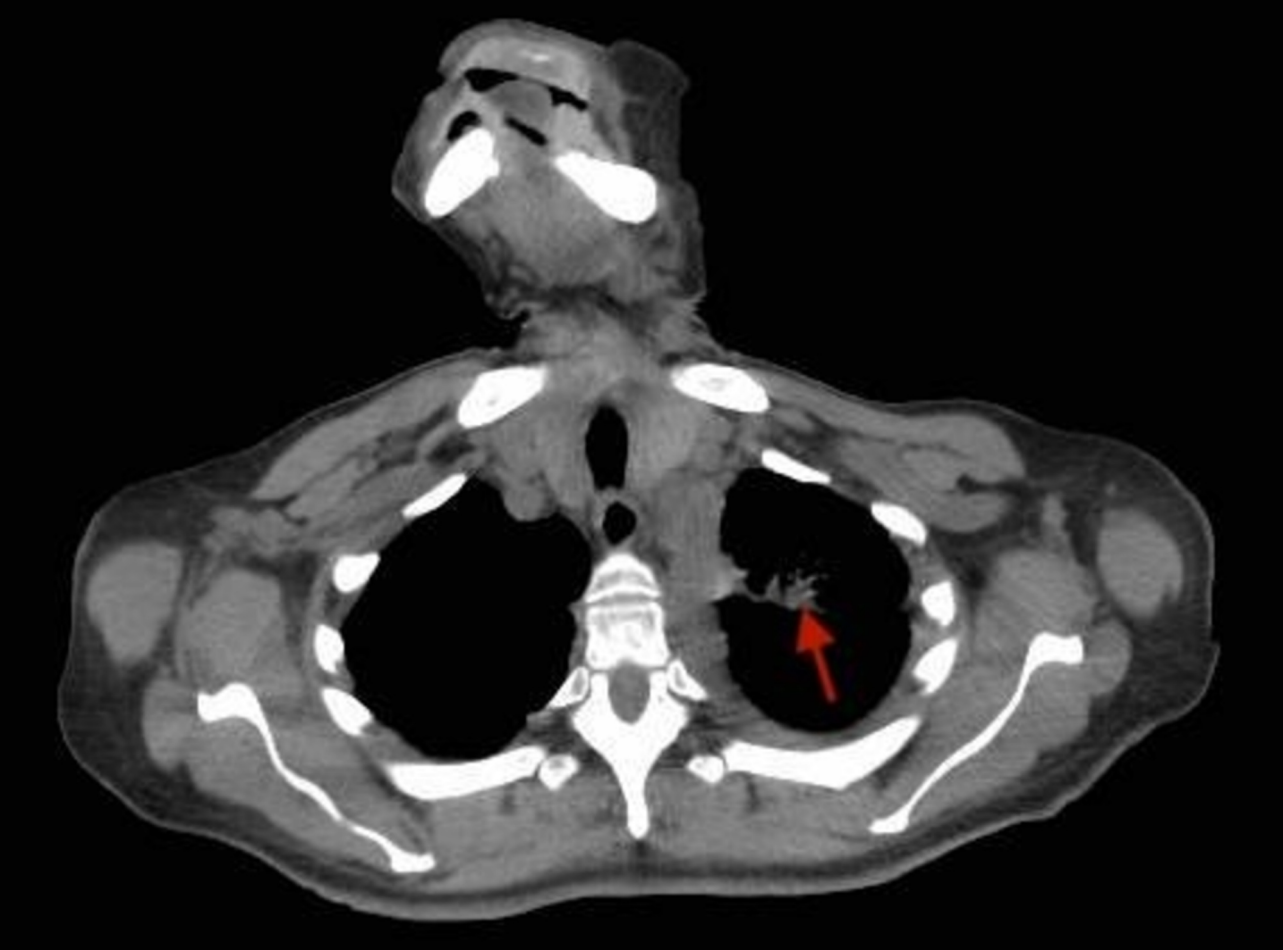
Axial CECT (contrast-enhanced computed tomography) scan of the chest showing a left lung mass extending into the upper lobe (red arrow).

An 80-year-old African American female with a 140-pack-a-year smoking history presented with complaints of chronic dry cough, dyspnea on exertion, hoarseness of voice, and an unintentional weight loss of 20 lbs over six months. Her past medical history was significant for multiple comorbid conditions, including hypertension, hyperlipidemia, and chronic renal failure. On general physical examination, she appeared visibly fatigued and experienced labored breathing. Her respiratory examination revealed reduced left-sided chest expansion, dullness to percussion, and decreased breath sounds over the left hemithorax. An electrocardiogram (EKG) was suggestive of left atrial enlargement and premature ventricular complexes. A chest radiograph showed a left hilar mass and complete opacification of the left hemithorax with a rightward tracheal deviation. These findings necessitated a follow-up with a contrast-enhanced computed tomography scan (CECT) of the chest, which revealed a large heterogeneous mass located in the left upper lobe. It was undifferentiated from the underlying atelectatic lung parenchyma and found to be obstructing the left upper lobe bronchus (Figures 1-4 show the CECT findings at the time of presentation). There was a loss of cleavage plane between the left lung mass and aorta. A 1.8 x 1 cm nodule (possible metastatic foci) was also visualized in the right upper lobe. Other pertinent findings included massive mediastinal lymphadenopathy (anterior mediastinal lymph node of size 2.7 x 2.2 cm), bilateral hilar lymph node enlargement, a small left-sided pleural effusion, and a moderate-sized pericardial effusion. CECT of abdomen and pelvis showed bilateral adrenal gland enlargement (left 3.1 x 1.5 cm, right 2.3 x 1.2 cm), and scattered paraaortic and pelvic lymphadenopathy suggestive of metastatic disease. No suspicious osseous lesions were identified. Bronchial brushing followed by cytology revealed atypical small blue cells in isolation and in small clusters, suggestive of malignancy. Differentials at that time included small-cell lung carcinoma vs. lymphoid malignancy. Lab work was done to assess the prognosis and rule out any paraneoplastic syndrome. Her lactate dehydrogenase (LDH) and alkaline phosphatase (ALP) levels were elevated (LDH: 673 IU/L, ALP: 208 IU/L). Serum calcium, total protein levels, and osmolality were normal, thus ruling out common paraneoplastic syndromes. The left-lung biopsy returned positive for small blue malignant cells (roughly twice the size of lymphocytes) with sparse cytoplasm and finely dispersed chromatin without any distinct nucleoli, confirming the diagnosis of small-cell lung carcinoma. Given the characteristic histopathology and distant metastases, a diagnosis of ED-SCLC was made. Systemic chemotherapy with etoposide/cisplatin (EP) was started in September 2015, and she underwent six cycles till April 2016. As the brain MRI was negative for metastasis, she received 25 Gy/10 fractions of prophylactic cranial irradiation (PCI).

**Figure 5:**
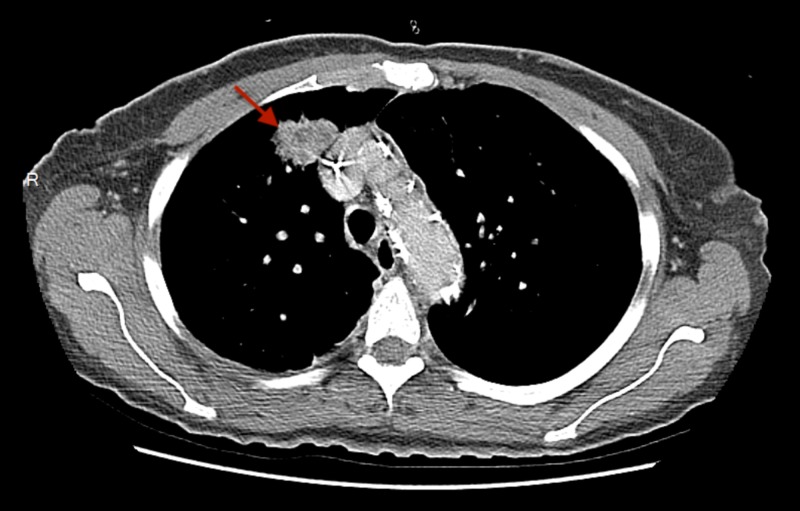
Axial CECT (contrast-enhanced computed tomography) scan of the chest showing a mass in the basal part of the right upper lobe (red arrow).

**Figure 6:**
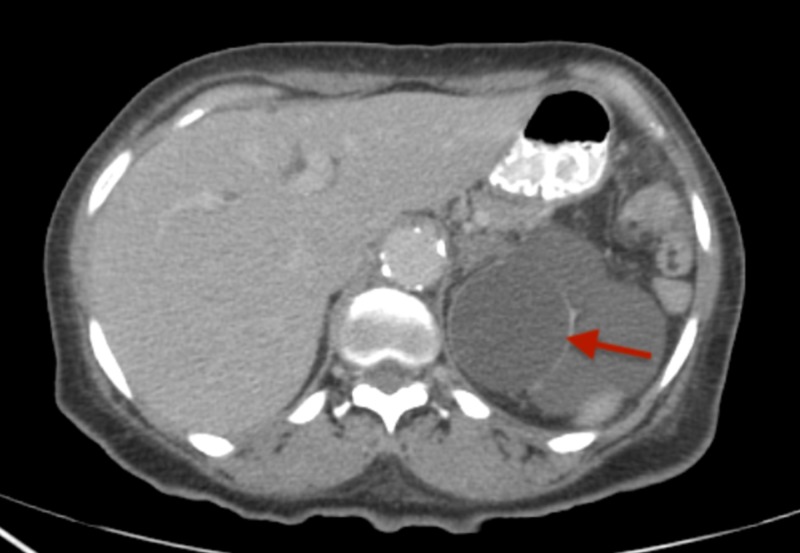
Axial CECT (contrast-enhanced computed tomography) scan of the abdomen showing left adrenal metastasis (red arrow).

**Figure 7:**
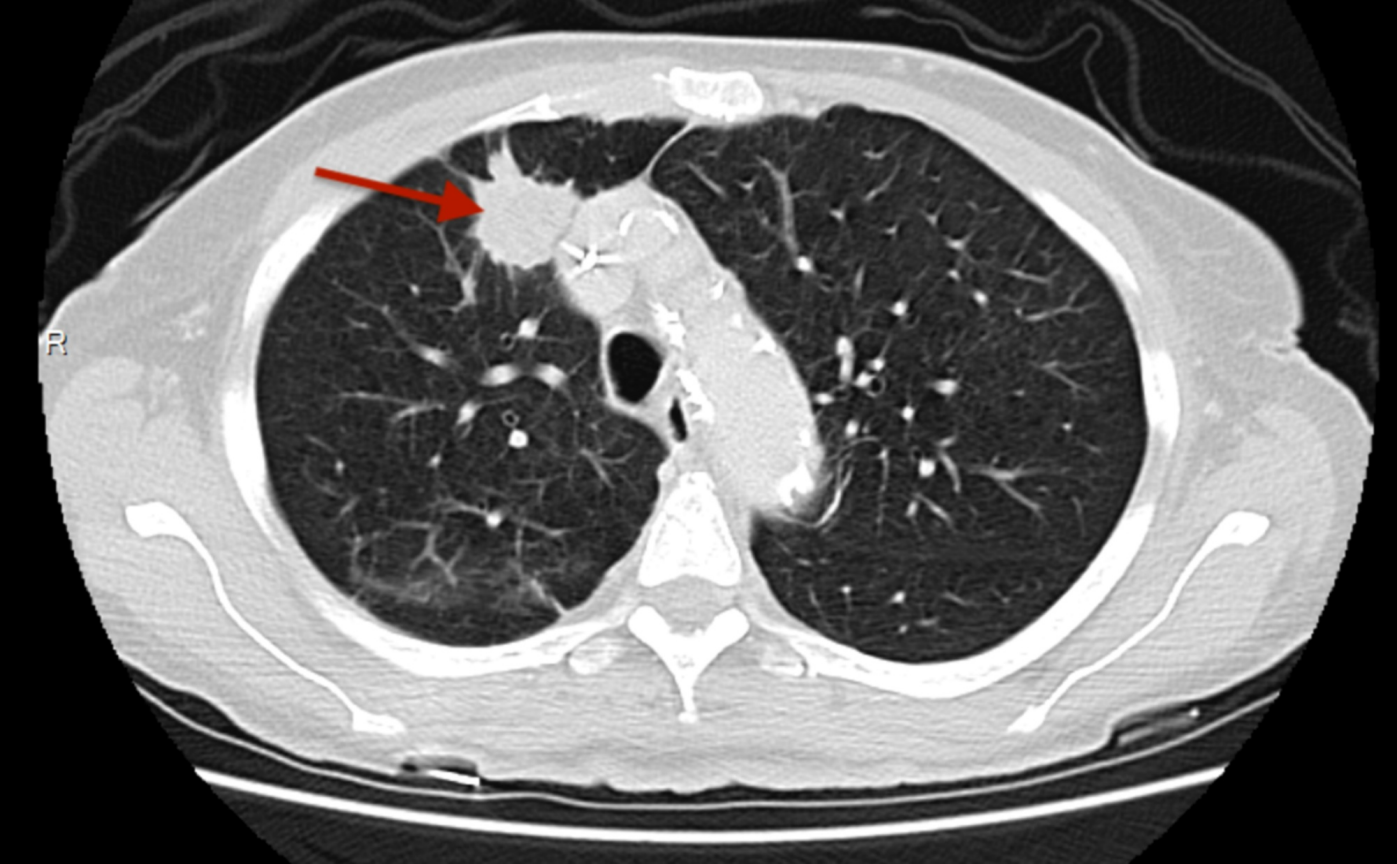
Axial CECT (contrast-enhanced computed tomography) scan of the chest showing a mass in the basal part of the right upper lobe (mass is slightly bigger in size as compared to Figure 5).

A positron emission tomography (PET)-CT scan after six cycles of EP showed an increase in the size of the lung nodules. Hence, five cycles of second-line chemotherapy with docetaxel were given from April-October 2016. She achieved partial remission, but a subsequent PET-CT after five cycles of docetaxel suggested an increase in the size of the right lung nodule. No follow-up was done on the patient from October 2016 to March 2017. She again presented in March 2017 with worsening dyspnea requiring continuous supplemental oxygen. A repeat CECT scan of chest and abdomen documented disease progression (Figures 5, 6). She was administered 12 cycles of irinotecan from March-October 2017. A CECT scan of the chest in October 2017 showed an increase in the size of the right lung nodule (Figure 7). Given the resistant nature of the disease, immunotherapy with 13 cycles of nivolumab was administered from October 2017 to July 2018. Ipilimumab was later added, but combination immunotherapy could not be continued beyond two cycles as the patient developed severe bloody diarrhea requiring hospitalization. Her hospital course was complicated by acute hypercapnic respiratory and supraventricular tachycardia, culminating in a cardiac arrest. As the patient was "do not resuscitate/do not intubate" (DNR/DNI), she could not be resuscitated and died 36 months after the diagnosis of SCLC.

## Discussion

The 2015 World Health Organization (WHO) classification describes four distinct histological variants of NE tumors of the lung: typical carcinoid (TC), atypical carcinoid (AC), large cell neuroendocrine carcinoma (LCNEC), and SCLC [1]. SCLC is the most common of NE tumors (20% of total lung cancers) [2]. TC and AC are relatively slow-growing tumors and generally have favorable outcomes. LCNEC and SCLC are both poorly differentiated tumors known for their dismal prognosis. They exhibit growth patterns that range from trabecular to solid to diffuse, extensive/geographic necrosis, and a mitotic count higher than 10 mitoses/2 mm^2^ with no theoretical upper limit [1].

The differential diagnosis of SCLC includes pulmonary carcinoid tumors (typical/atypical) and other rare neuroendocrine lung tumors. When compared to carcinoids, SCLCs usually affect older patients (mean age of 65 years), are more strongly associated with smoking, have a greater tendency to metastasize, and are treated with combined chemo-radiotherapy due to higher sensitivity (limited-disease variant) [2-4, 5]. Atypical carcinoid tumors are usually situated along the lung periphery, while SCLCs are often more central in location. Other important differentiating factors include a higher chromogranin A reactivity in carcinoid tumors and a more prominent TTF-1 staining and Ki-67 proliferative assay in SCLC [6].

A two-stage system developed by the Veteran's Administration Lung Cancer Study Group (VALG) in 1957 is widely used in the staging of SCLC. It classifies the patients into two distinct categories: limited disease (LD) and extensive disease (ED). A patient is placed in the former category when the tumor is confined to one hemithorax and its regional lymph nodes, including the ipsilateral mediastinal, ipsilateral supraclavicular, and contralateral hilar lymph nodes. Tumors that present with ipsilateral pleural effusion, left laryngeal nerve involvement, or superior vena cava obstruction are also placed under the LD category. ED, on the other hand, comprises all patients with sites of disease beyond the definition of LD. When compared with the more organized TNM method of classification, ED is equivalent to stage IV, whereas LD is equivalent to stage I-III of the revised TNM system [7]. The five-year survival rate of limited disease small-cell lung cancer (LD-SCLC) and ED-SCLC are 10-13% and 1-2% respectively. Less than 5% of patients with ED-SCLC survive for two years. The presence of ED, involvement of multiple organ sites (especially the central nervous system, the marrow, or the liver), elevated LDH levels, weight loss, ectopic hormone production, poor performance status, advanced age, African-American race, poor tolerance and response to initial treatment, and early relapse are considered poor prognostic factors in the setting of SCLC [8].

The National Comprehensive Cancer Network (NCCN) and the European Society for Medical Oncology (ESMO) panels have adopted a combined approach for staging SCLC, which incorporates both the TNM staging system as well as the VALG protocol for SCLC. This approach suggests that the methods of assessment and treatment planning should be based on the two-stage system, while the choice of surgery and radiotherapy should be based on the TNM system. Chemo-radiotherapy is the backbone of the treatment of SCLC. NCCN guidelines recommend that patients with LD-SCLC (T1-4 N0-3, M0) and an Eastern Cooperative Oncology Group performance status (PS) of 0-2 should receive concurrent chemo-radiotherapy with radiotherapy starting possibly with the first or second chemotherapy cycle. Also, etoposide and carboplatin/EP/irinotecan and cisplatin/irinotecan and carboplatin can be used as first-line chemotherapy drugs [8]. Radiotherapy may be delayed in LD patients who are not fit for concurrent chemo-radiotherapy [9]. In patients with ED, chemotherapy is started first, and the need for sequential thoracic radiotherapy is determined based on the response to chemotherapy. For example, radiotherapy may be considered for selected patients with the residual intrathoracic disease and low metastatic burden [8].

At least 20% of SCLC patients present with brain-tissue involvement at the time of diagnosis. Even in patients who achieve a complete response to treatment, there is a 45% chance of recurrence in the brain. Reasonably, PCI should be considered for LD-SCLC and ED-SCLC patients who achieve disease control after the completion of the initial treatment regimen. The highest total radiotherapy dose recommended by the NCCN is 25 Gy in 10 daily sessions. Even though neurotoxicity is a major concern, results from retrospective trials have shown benefit from PCI over chronic toxicity [10].

SCLC is usually very responsive to initial treatment. However, most patients relapse with relatively resistant disease and require second-line chemotherapy. The likelihood of response is contingent upon variables like the time from the response to initial therapy to relapse and performance status of the patient. NCCN recommends that patients with a performance status of 0-2 who relapse within six months after treatment may be treated with topotecan (PO or IV), irinotecan, paclitaxel, docetaxel, temozolomide, vinorelbine, oral etoposide, gemcitabine, cyclophosphamide/doxorubicin/vincristine, bendamustine, or nivolumab with/without ipilimumab. Patients who relapse six months after treatment can be treated with the original regimen [8].

Immunotherapy with checkpoint inhibitors has already been included in the NCCN guidelines. Checkpoint inhibitors have proven their efficacy in other solid tumors that are associated with a high non-synchronous mutation burden, a feature consistent with SCLC [11]. Currently, specific inhibitors targeting two main checkpoints (the cytotoxic T lymphocyte antigen-4 [CTLA-4] receptor and the programmed death-1 [PD-1] receptor) and the ligands of the PD-1 receptor (programmed death-ligand 1 [PD-L1] and programmed death-ligand 2 (PD-L2]) are available. Ipilimumab and tremelimumab are two monoclonal antibodies that target the CTLA-4 receptor, and they are currently under investigation. The anti-PD-1 drugs nivolumab and pembrolizumab are already in the advanced stages of clinical development. Several ongoing trials are exploring the role of immunotherapy as a first-line treatment in ED-SCLC patients. No data is currently available regarding the efficacy of immunotherapeutic agents in LD-SCLC patients [12]. These drugs come with their fair share of side effects, which may vary from trivial pruritus and maculopapular rash to life-threatening colitis, pneumonitis, hepatitis, nephritis, and encephalitis. Patients often require close monitoring while on these drugs. If a patient develops one of the deleterious side effects, immunotherapy should be discontinued, and corticosteroids should be started promptly. The re-initiation of the treatment depends on the severity of the condition [13].

## Conclusions

SCLC is a disease known for its abysmal prognosis. However, there are surprisingly encouraging reports of a small group of patients with prolonged survival time. Our report discusses a case of a long-term survivor of SCLC who was treated with radiotherapy and several lines of chemotherapy. But the progress in the therapy of SCLC has generally been very slow. To tackle this dreadful disease, a better understanding of its biology is imperative so that more effective therapies can be developed. There is also a need to establish guidelines that would aid in the selection of SCLC patients with a favorable prognosis so that optimal therapeutic approaches may be devised for such patients on a case-by-case basis.
